# Optimizing School Food Supply: Integrating Environmental, Health, Economic, and Cultural Dimensions of Diet Sustainability with Linear Programming

**DOI:** 10.3390/ijerph16173019

**Published:** 2019-08-21

**Authors:** Patricia Eustachio Colombo, Emma Patterson, Liselotte Schäfer Elinder, Anna Karin Lindroos, Ulf Sonesson, Nicole Darmon, Alexandr Parlesak

**Affiliations:** 1 Department of Public Health Sciences, Karolinska Institutet, 171 77 Stockholm, Sweden; 2 Centre for Epidemiology and Social Medicine, Stockholm County Council, 112 21 Stockholm, Sweden; 3 The National Food Agency, Uppsala, Sweden and Department of Internal Medicine and Clinical Nutrition, Institute of Medicine, Gothenburg University, 405 30 Gothenburg, Sweden; 4 RISE Research Institutes of Sweden, 420 29 Gothenburg, Sweden; 5 MOISA, INRA, Univ Montpellier, CIHEAM-IAMM, CIRAD, Montpellier SupAgro, 34060 Montpellier, France; 6 Global Nutrition and Health, University College Copenhagen, 2200 Copenhagen, Denmark

**Keywords:** nutrition, children, greenhouse gas emissions, school meals, sustainability, Agenda 2030

## Abstract

There is great potential for reducing greenhouse gas emissions (GHGE) from public-sector meals. This paper aimed to develop a strategy for reducing GHGE in the Swedish school food supply while ensuring nutritional adequacy, affordability, and cultural acceptability. Amounts, prices and GHGE-values for all foods and drinks supplied to three schools over one year were gathered. The amounts were optimized by linear programming. Four nutritionally adequate models were developed: Model 1 minimized GHGE while constraining the relative deviation (RD) from the observed food supply, Model 2 minimized total RD while imposing stepwise GHGE reductions, Model 3 additionally constrained RD for individual foods to an upper and lower limit, and Model 4 further controlled how pair-wise ratios of 15 food groups could deviate. Models 1 and 2 reduced GHGE by up to 95% but omitted entire food categories or increased the supply of some individual foods by more than 800% and were deemed unfeasible. Model 3 reduced GHGE by up to 60%, excluded no foods, avoided high RDs of individual foods, but resulted in large changes in food-group ratios. Model 4 limited the changes in food-group ratios but resulted in a higher number of foods deviating from the observed supply and limited the potential of reducing GHGE in one school to 20%. Cost was reduced in almost all solutions. An omnivorous, nutritionally adequate, and affordable school food supply with considerably lower GHGE is achievable with moderate changes to the observed food supply; i.e., with Models 3 and 4. Trade-offs will always have to be made between achieving GHGE reductions and preserving similarity to the current supply.

## 1. Introduction

The emission of anthropogenic (human-induced) greenhouse gases has been established as a driver of climate change. It is one of three earth system processes that has reached critical levels [1] and is therefore a major threat to the health of humans, animals, and natural habitats [2,3]. Today’s food production systems account for about 25% of the world’s anthropogenic greenhouse gas emissions (GHGE), and contribute substantially to deforestation, the exploitation of land and freshwater, nitrogen cycle disruption, and the loss of biodiversity [4]. Increasing wealth and urbanization often result in a dietary shift towards increased consumption of resource-demanding and greenhouse gas-intensive foods of animal origin, and a reduced consumption of low-processed plant-based foods such as legumes, vegetables and fruits [5,6,7]. Moreover, nutritionally inadequate diets have been shown to have negative impacts on both mortality and morbidity from non-communicable diseases such as cardiovascular diseases, cancer and type-2 diabetes [8]. Hence, in order to improve health, reduce anthropogenic GHGE, and further contribute towards reaching several of the 2015 Sustainable Development Goals [9] and the Paris Agreement [10], fundamental changes of our diets are needed, as also emphasised by the 2019 EAT-Lancet Commission on healthy diets from sustainable food systems [11].

In 2012, the Food and Agriculture Organization (FAO) of the United Nations (re-)established the concept of sustainable diets and described them as: “[…] those diets with low environmental impacts which contribute to food and nutrition security and to healthy life for present and future generations. Sustainable diets are protective and respectful of biodiversity and ecosystems, culturally acceptable, accessible, economically fair and affordable; nutritionally adequate, safe and healthy; while optimizing natural and human resources” [12]. These dimensions of diet sustainability are not always compatible or synergistic, and often trade-offs have to be made between different demands such as nutritional adequacy, environmental impact, and affordability [13,14,15,16,17,18,19,20].

One method suitable for optimizing diets and mathematically identifying the best trade-offs is linear programming. Some studies in the area of diet sustainability optimization have used this method [14]; more common are studies that explore the concept by assessing health and/or environmental aspects of self-selected diets [13,15,16,17,18,19]. However, optimization has been used to minimize the cost of nutritionally adequate diets [20,21,22], as well as to identify diets with reduced GHGE [23,24,25,26,27,28]. To the best of our knowledge, no study has yet applied the method to reducing GHGE while planning public meals.

In Sweden, meals produced and served in the public sector such as in schools, hospitals, and care homes are provided to up to one third of the population (about three million people) daily. Fully subsidized lunches are served daily in primary schools to all 1.3 million children aged 6 to 15 [29]. Due to their reach and scale (approximately 230 million/year), school meals have considerable potential to shape children’s diets and reduce food-related GHGE—in both the short and long term. GHGE attributed to food consumption per person in Sweden has been estimated to be about two tons of carbon dioxide equivalents [30].

The aim of this study was to find an appropriate strategy of reducing GHGE in the Swedish school food supply using linear programming, while ensuring its nutritional adequacy, affordability, and cultural acceptability.

## 2. Materials and Methods

### 2.1. Data Acquisition

#### 2.1.1. Annual Observed School Food Supply

Three municipalities in Sweden provided data on school food purchases for one of their primary schools. Information on all foods and drinks purchased during the school year of 2015/2016 was obtained through the municipality’s procurement system [31]. This system provides data on amounts of each food bought in kilograms (kg) and their price (total cost and price per kg). The weight of nutritionally identical food items, bought on several occasions over the school year, was aggregated to a total weight for each school separately. Foods bought as organic and non-organic variants, as well as frozen and fresh, were not aggregated, but instead treated as separate food items due to differences in price. The price of each food was calculated based on the average price paid for all deliveries of that food weighted by the amount ordered. Very expensive foods and drinks contributing only marginally to nutrient supply such as spices, foods for special needs (e.g., gluten-free bread), bottled water, baking powder, or items considered to have been bought for canteen staff such as coffee and tea were excluded from the list. In the end, the observed food supply was based on 499 food items in School 1, 539 items in School 2, and 367 items in School 3. The variation in the number of foods was partly based on the number of organic foods used, which were counted as separate food items.

The National Food Agency’s guidelines for “Good school meals” recommends that a school lunch should on average provide 30% of the daily dietary reference values [32]. Assuming a school with 50% girls, 50% boys, and equal numbers of pupils in each of the 10 primary school age categories (6–15 years), a reference lunch for a reference pupil should provide 604 kilocalories (kcal) according to these recommendations. This value, together with the total amount of kcal for all foods purchased by each school for the entire school year, was used to calculate an energy-standardized food supply for one reference pupil and lunch; i.e., the energy-proportional shares of each food item adjusted for the estimated energy requirement. The energy-proportional shares of each food for one pupil and lunch were calculated for modelling purposes and represent the observed food supply for the entire school year. For example, all foods purchased for the entire school year in School 1 contained approximately 46 million kcal. The total amount of salmon purchased was 99 kg, with an energy content of approximately 99 thousand kcal. As the energy content for a lunch for a reference pupil should be 604 kcal, the energy-proportional share of salmon for one pupil and lunch was about 1.3 g (i.e., the average intake per day over the school year). This approach was applied to all foods, which together constituted the observed food supply per pupil and lunch.

#### 2.1.2. Nutritional Composition of Foods

Data on the nutritional composition of foods as eaten (e.g., cooked rice) were extracted from the Swedish National Food Agency’s food database containing 2088 food items [33]. For foods not appearing in this database, data from the Norwegian Food Composition Table [34] and the USDA food composition databases [35] were consulted, respectively. Yield factors and edible proportions, as provided by the food composition databases, were applied to convert weights of purchased raw foods into weights of edible food. Foods delivered in units (e.g., limes) were converted to weights [36]. All calculations of nutritional adequacy referred to the nutrient content of the edible shares of prepared (cooked, simmered, fried, baked, etc.) foods (see below). Thus, unavoidable kitchen food waste is considered in all calculations. Although nutritional adequacy was calculated based on the composition of edible shares, the results (cost, weight, GHGE) refer to the amount of raw food as purchased. The salt intake was estimated to be 20% of the purchased amount based on the estimation that only a part of the salt applied to cooking water ends up in consumed food such as pasta [33,34].

#### 2.1.3. Greenhouse Gas Emissions (GHGE) of Foods

The GHGE of the foods were expressed as carbon dioxide equivalents (CO_2_eq) of food products. The specific data were extracted from the Climate Database from the Research Institutes of Sweden (RISE), which builds on results from life cycle analyses [37,38] and typical Swedish food supply/purchasing patterns [39]. Differences according to production systems, origin, assumed mode of transportation and consumption are thereby accounted for. For example, the GHGE emission for the average tomato consumed in Sweden is the average GHGE for tomatoes grown in Sweden, the Netherlands and Spain, weighted by share of total consumption. The database contains values for several GHGE (carbon dioxide, CO_2_; methane, CH_4_; and nitrous oxide, N_2_O) that are weighted in line with their respective global-warming potential over a 100 year period, using factors recommended by the Intergovernmental Panel on Climate Change [40]. This yields a single value for the combined GHGE, measured as kg of CO_2_eq per kg of food item (kg CO_2_eq/kg), also known as “carbon footprint”. The system boundaries for calculating the CO_2_eq values are from primary production until the factory gate (packaging, further distribution to shops and homes, meal preparation after delivery, and waste management are not included). The database contains 2078 foods commonly consumed in Sweden as well as foods of particular interest from a nutritional and/or environmental point of view and is linked to the Swedish National Food Agency’s food database with information on nutritional content.

As the nutritional value of the supply was calculated based on edible shares but the CO_2_eq value was based on the weight of the raw food, the reported CO_2_eq took unavoidable food waste into account. For analytical and descriptive purposes, foods were grouped in 15 food categories, based on the climate database (cereals, bread, solid dairy (e.g., cheese), other dairy (e.g., milk), red meat (including offal), poultry, pulses, roots and tubers, vegetables (e.g., tomatoes, cucumber, lettuce), fruits and berries, fish, oils, solid fats (e.g., butter, margarine), eggs, and other (e.g., seeds, salt, sugar, jams)).

### 2.2. Optimization

#### 2.2.1. Linear Optimization

Linear programming (LP) is the application of an algorithm to maximize or minimize a given (linear) objective function subjected to a set of (linear) constraints on a list of decision variables [41]. It consists of three major elements: (i) the objective function (a loss function or its negative of the goal variable), (ii) the decision variables (the variables to be changed by the model), and (iii) a set of constraints (criteria to be met). If all conditions can be met, then a solution is said to be found. In LP models, constraints that determine the degree to which the objective function can be minimized or maximized are called “active constraints” [42]. Nutrients that met exactly 100% of their reference values in the solution were identified as active constraints. Non-active constraints are per definition above the minimum or below the maximum limit, once the active constraints have determined the solution of the model. Linear optimization was performed with the CBC (Coin-or branch and cut) Solver algorithm, which is part of the Excel^®^ 2016 software add-in OpenSolver, V. 2.8.6 [43].

#### 2.2.2. Nutritional Adequacy of Optimized Food Supply

Based on the Nordic Nutrition Recommendations 2012, dietary reference values (DRVs) for planning school meals in Sweden were implemented as obligatory constraints in the optimizations [44,45]. The DRVs used were the equivalents (30%) of the daily estimated energy requirements (EER), recommended intake ranges for macronutrients, and the recommended intakes (RIs) and/or estimated upper intake levels (ULs) for micronutrients [45]. The nutritional constraints for a reference pupil were calculated by averaging the DRVs over ten ages and both sexes (Table S1). All optimized food supply solutions met the DRVs for an average Swedish school lunch.

After optimization, the amounts of the supply of the optimized food groups were compared to the recommended intakes. For this comparison, the Swedish food-based dietary guidelines (FBDGs) were used [46].

#### 2.2.3. Total GHGE of Observed and Optimized Food Supply

The GHGE of the observed and optimized food supply were calculated as the sum of the corresponding raw food weights multiplied by their specific CO_2_eq value in the Climate Database.

#### 2.2.4. Total Cost of Observed and Optimized Food Supply

The total weight of each food product was calculated and multiplied by the specific cost of the product as purchased by the schools to obtain the cost of the observed and optimized food supply, respectively.

#### 2.2.5. Deviation from Observed Food Supply

The similarity of the optimized supply with the observed supply is determined by the relative deviation of individual foods after optimization compared to that before. For this purpose, the relative (i.e., percentage) deviation (RD) for each food was calculated. For example, if 100 kg of potatoes were used in the previous school year in one of the school canteens and 120 kg of potatoes were included in the optimized solution, the RD would be +20%. Hence, the absolute relative deviation [abs(RD)] of the amounts suggested by the linear programming algorithm from the observed supply of each food item is the non-negative value of the relative deviation and was calculated for each food item according to Formula (1):

(1) $$\mathrm{abs}\left({\mathrm{RD}}_{i}\right)=\frac{\mathrm{abs}\left(M_{i}-m_{i}\right)}{m_{i}}$$

where m_i_ stands for the observed supply of the i-th food item in grams provided to the reference pupil and M_i_ is the weight of the i-th food item after optimization. The absolute value of RD for each individual food item [abs(RD_i_)] was used as a constraint in Models 1, 3 and 4. In order to achieve the least deviation from (or the highest similarity to) the observed food supply—the total sum of the absolute values of RDs—the total relative deviation (TRD) from all N food items in the model was calculated and used as the objective function in Models 2, 3 and 4 (Formula (2)):

(2) $$\mathrm{TRD}\;=\;\sum_{i=1}^{N}\mathrm{abs}\left({\mathrm{RD}}_{i}\right)$$

The average relative deviation (ARD) from the observed food supply was used as a proxy of similarity between the observed and the optimized food supply and was calculated by dividing the TRD by the total number of food items included in the model (N), as given in Formula (3):

(3) ARD=TRD/N

Previous studies have considered the preservation of ratios of foods or food groups—for example, jam to bread—to be an important factor for the acceptability of optimized diets [47,48,49,50]. The absolute (non-negative) value of each relative ratio deviation of the optimized to the observed food group [abs(RRD)] can be used as a measure for similarity. The abs(RRD) can be calculated according to Formula (4):

(4) $$\mathrm{abs}\left({\mathrm{RRD}}_{j}\right)=\frac{\mathrm{abs}\left(R_{j}-r_{j}\right)}{r_{j}}$$

where r stands for the observed supply of the j-th food-group and R_j_ is the supply of the same food group after optimization. The average (non-negative) relative ratio deviation (ARRD) from the observed food supply was used as another proxy of similarity between the observed and the optimized food supply and was calculated by dividing the sum of absolute RRDs by the model’s total number of food-group ratio pairs (105 in total), as given in Formula (5):

(5) $$\mathrm{ARRD}=\frac{\left(\sum_{j=1}^{N}\mathrm{abs}\left({\mathrm{RRD}}_{j}\right)\right)}{M}$$

In Formula (5), M stands for the number of possible food-group ratios (105 in total).

#### 2.2.6. Models

In this study, four different models were developed for the LP analyses. An overview of the applied models and their corresponding objective functions, constraints and calculated outputs is given in Table 1.

#### 2.2.7. Model 1: Minimizing the GHGE of the Observed Food Supply While Meeting Nutritional Constraints (GHGE_min_)

In Model 1, the objective function of the linear programming model was the minimization of the total GHGE (calculated as CO_2_eq) of the observed food supply. The decision variables were the amounts of edible foods that were eligible to be included into the optimized food supply for one pupil and one lunch. The only set of constraints initially applied was to meet the DRVs of energy and nutrients to see how much GHGE could be reduced while ensuring nutritional adequacy. Each food item was allowed to increase or decrease unconditionally from the observed food supply. Next, the RD of each food item was constrained in a step-wise process in order to limit the deviation from the observed food supply. Each food was allowed to increase/decrease first by +1000/−100%, then by +500/−100%, +300/−100%, ±100%, ±99%, ±90%, ±80%, ±70%, ±60%, ±50%, ±45%, and ±40% or until the model did not provide any feasible solution (i.e., one that met all applied constraints). This was done to (i) explore how the school food supply changed compared to baseline for each step of restriction, (ii) to explore the changes occurring between the different steps, and (iii) to investigate whether using GHGE as the goal function (in contrast to TRD) provides reasonable solutions. The computed outputs of the model were the TRD, the ARD, the cost and the total daily GHGE. As the TRD depended on the original number of foods in the school’s observed food supply, the TRD was not comparable across the schools. Therefore, only the ARD value is reported.

#### 2.2.8. Model 2: Minimizing the TRD from the Observed Food Supply with a Stepwise Reduction of GHGE (TRD_min_)

Model 2 was developed with the aim of attaining a higher degree of similarity to the observed food supply (i.e., less deviation) than that achieved in Model 1, but with comparable GHGE reductions. In Model 2, the objective function of the LP model was the minimization of the TRD from the observed food supply while still ensuring nutritional adequacy and imposing stepwise reductions of GHGE by relative values until a feasible solution could not be found. As TRD is not a linear function and therefore cannot be part of the linear equation system used by LP, new decision variables Z_i_: Z_1_→Z_n_ were created according to Darmon et al. [51]. The new decision variables were submitted to the following constraints (Formula (6)):

Z_i_ ≥ (m_i_ − M_i_)/m_i_ and Z_i_ ≥ −(m_i_ − M_i_)/m_i_ (6)

Thus, for each standardized difference, its absolute (positive) value was selected because Z_i_, by definition, has to be greater than or equal to both the relative difference and its inverse value. The TRD_min_ model allowed for the minimization of the sum of the absolute values of all relative deviations from the observed food supply [51]. In the TRD_min_ model, no limits were set to the RDs of the individual food items supplied or the changes in food-group ratios (abs(RRD_j_)). The computed outputs of Model 2 were the total daily GHGE, the ARD, the average relative ratio deviation (ARRD), the cost, and the number of foods removed, reduced or increased compared to the observed food supply. For selected solutions, the relative change in the total weight of the food groups (RFGC) was visualized and calculated as the relative change between the observed and the optimized weights in each food group.

#### 2.2.9. Model 3: Minimizing the TRD from the Observed Food Supply with a Stepwise Reduction of GHGE While Constraining the RD of Individual Food Items to Range between an Upper (Positive) and a Lower (Negative) Limit (CTRD_min_)

In Model 3 (CTRD_min_), we limited the RD of individual food items to decrease or increase by an upper (positive, indicating maximum relative increase) and a lower (negative, indicating maximum relative reduction) value (one positive and one negative value) in order to avoid the extreme deviations of individual food items from the observed supply, as provided by Model 2. The computed outputs of the model were the total daily GHGE, the ARD, the ARRD, the cost, and number of foods removed, reduced or increased from the observed food supply. For selected solutions, the RFGC was visualized and calculated as the relative change between the observed and the optimized weights in each food group.

#### 2.2.10. Model 4: Minimizing the TRD from the Observed Food Supply with a Stepwise Reduction of GHGE While Constraining Both RDs of Individual Food Items and Ratios between Food Groups to Range between an Upper and a Lower Limit (RTRD_min_)

In Model 4 (RTRD_min_), we limited the RD of individual food items as in Model 3 and added constraints on the deviation of all food-group ratios (RRDs). Ratios between foods or food groups are non-linear functions and can therefore not be directly implemented as constraints in linear programming. Differences between relative changes of food groups (RFGDs) (observed vs. optimized) can, however, be implemented. The smaller the RFGD allowed, the smaller the occurring changes in ratios between food groups. For example, allowing 30% for the RFGD meant that if one food group decreased by 20%, the other food group could not increase by more than 10%, which constrained their ratio to a value between 0.73 and 1.33. The RFGD (Formula (7)) was constrained by steps of 10% for reductions of GHGE of 20%, 30%, and 40%.

(7) $$\mathrm{RFGD}=\;\mathrm{abs}\left[\frac{\left(R_{j}-r_{j}\right)}{r_{j}}-\frac{\left(R_{k}-r_{k}\right)}{r_{k}}\right]$$

where R stands for the amount of the optimized supply of one of the 15 food groups listed under “Greenhouse gas emissions of foods” and r indicates the observed supply. The indices j and k indicate the different food groups, so j may indicate for example “solid fats” and k may indicate “bread”. The RFGD constraint was applied to all possible combinations of the 15 food groups (105 in total).

The outputs of the model were the ARD, the ARRD, the cost, and the total daily GHGE. Additional outputs of all models were the type and number of foods removed, reduced or increased from the observed food supply. The RFGC was visualized for some solutions and calculated as the relative change between the observed and the optimized weights in each food group.

## 3. Results

Initially, the observed food supplies for the three schools, when standardized to an energy requirement of 604 kcal per pupil and lunch, were associated with GHGE of 810 g, 1022 g, and 967 g CO_2_eq, at a cost of 9.1, 10.6, and 11.2 Swedish krona (SEK), respectively (1 SEK ≈ 0.104 United States dollar or 0.092 €). The observed food supply did not meet the requirements for vitamin D (nutrient supply was 61–97% of RI), iron (82–88% of RI), and saturated fatty acids (135–140% of %E targets).

Model 1 (GHGE_min_) reduced GHGE by 89–95% (GHGE values between 51 and 86 g/meal) but contained only 7–9 foods, and the ARD from the observed food supply was 480–887% (Tables S2 and S3). The model resulted in radical changes in food supply, where food groups such as red meat, eggs, and dairy were entirely omitted. When progressively limiting the maximum relative deviation (abs(RD)) in Model 1, the number of foods in the optimized supply increased, but so did the GHGE (Table S3).

In Model 2 (TRD_min_), comparable GHGE reductions (80–90%) at lower ARD (78–459%) (Table 2) were achieved but with a high relative increase of single foods (Figure S1). Although the ARD values in Model 2 were lower compared to Model 1 (at comparable GHGE reductions), the relative increase in the supply of some individual foods turned out to be high (Figure S1). For example, at 40% lower GHGE, bread in Schools 1 and 2 and offal in School 2 increased more than 8-fold.

**Figure 1:**
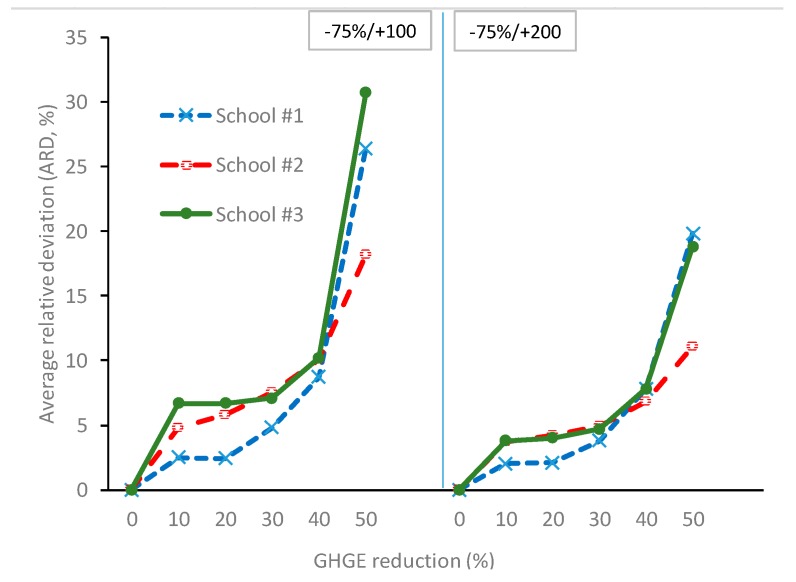
Average relative deviation (ARD) in relation to GHGE reduction (by steps of 10%) when minimizing total relative deviation (TRD) and applying constraints on nutritional adequacy, relative GHGE reductions, and additionally constraining the relative deviation (RD) of individual food items from observed food supply to a range between −75%/+100 and −75%/+200% (Model 3). GHGE, greenhouse gas emissions. The RD of the optimized solutions refers to the observed food supply during the school year 2015/2016.

**Figure 2:**
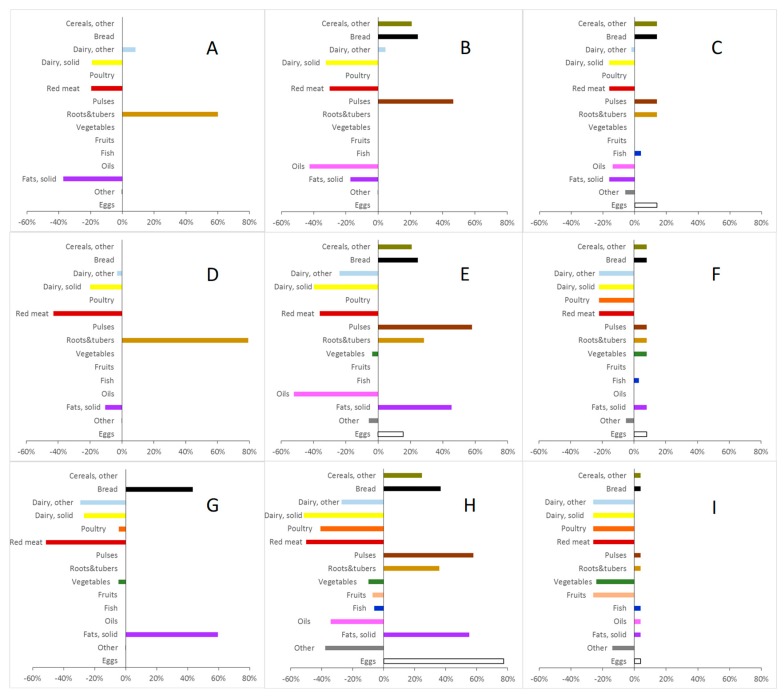
Effect of GHGE reduction (Panels **A**–**C**, −20%; **D**–**F**, −30%; **G**–**I**: −40%), limited relative food deviation (RD) (Panels **A**,**D**,**G**, unconstrained (Model 2); **B**,**E**,**H**, maximum RD: −75%/+100% (Model 3); Panels **C**,**F**,**I**, as Model 3 plus maximum allowed differences between relative changes of food groups (RFGDs) of 30% (Model 4) on relative changes of food groups in School 1. All provided solutions accommodate the nutritional guidelines for Swedish school meals [32].

In Model 3 (CTRD_min_), limiting the RD of individual food items from the observed food supply to an upper (positive) (+100% or +200%) and lower (negative) limit (−75%) resulted in more foods being reduced or increased compared to Model 2, where foods were allowed to be excluded entirely and/or to increase unconditionally (Table S4). As shown in Figure 1, the ARD in Model 3 did not increase markedly until a GHGE reduction of 30–40% was reached. At 40% lower GHGE, the ARD was slightly higher in Model 3 (6.8–10.2%) as compared to Model 2 (2.7–4.6%) (Table 2). However, the high relative increase in the supply of some foods in Model 2 (Figure S1) was avoided in Model 3 (Figure S2). Moreover, no foods were excluded entirely in Model 3, thus increasing the food variety of the optimized solutions. Compared to Model 2 (Figure 2A,D,G), the higher number of foods changed after limiting the RD in Model 3 (Table S4) was paralleled by a higher number of food groups changed (Figure 2B,E,H). Offal and milk remained in the optimized supplies while the amount of other red meat was reduced (Figures S1 and S2). Model 3 had ARRDs ranging between 55.3% and 95.0% at 40% lower GHGE (Table 2).

In Model 4 (RTRD_min_), adding constraints on RFGDs (between 50% and 0%) limited the ability to reduce the GHGE of the food supply as compared to the less restricted solutions from Models 2 and 3. For one of the schools, no solution was found for a GHGE reduction of more than 10% when applying Model 4 (Table 2). Applying constraints on RFGDs (and therefore ratios between food groups) resulted in a higher number of foods (Table S4) and food groups (Figure 2) being changed. In Model 3, about 40 foods were changed; in Model 4, up to more than 300 foods were changed (at 40% GHGE reduction, Table S4). Compared to Model 3 at −40% GHGE, the ARD values increased to 75.4%, meaning that the additional application of constraints on food-group ratios increased the deviation of the single foods from the observed supply considerably. However, at 40% GHGE reductions, the average relative ratio deviations (ARRDs) were lower in Model 4 (0–30.6) compared to Model 3 (55.3–95.0) (Figure 2, Table 2).

The cost of all optimized food supplies was either below the observed cost or exceeded the observed values only marginally, depending on the applied constraints (Model 1, SEK 3.9–10.9; Model 2, SEK 4.2–11.7; Model 3, SEK 7.8–11.3; Model 4, SEK 7.9–10.9) (Tables S3 and S4).

Based on data used for Figure 2H, the optimized food amounts from Model 3 (constraint of −75/+100 RD for individual foods), which was 40% lower in total GHGE, met the Swedish FBDG recommendations for fruit and vegetables (30% of 500 g/day), provided at least 30% of the recommended 2 portions of 130 g of fish/week, and no more than 30% of the maximum recommended 600 g red and processed meat per week in all schools.

## 4. Discussion

In this study, we have shown that considerable reductions in GHGE (up to 40%) could be achieved with only small changes to the observed school food supply while ensuring nutritional quality or cost. Minimizing TRD enabled a greater degree of similarity to the observed food supply than models where GHGE was minimized at comparable GHGE reductions. Solutions that used GHGE as a goal function (Model 1) or minimized the total relative deviation of the food supply without applying further measures of acceptability (Model 2) were judged to be unrealistic due to the omission of entire food categories and/or extreme changes in amounts of single foods. In contrast, when constraining how much individual foods could deviate (Model 3) and additionally preserving ratios between food groups (Model 4), more foods and food groups were changed, but their relative deviation from the observed food supply was compromised compared to preceding models. We therefore propose that TRD is a better choice as a goal function than GHGE, but that in order to achieve a high acceptability when planning meals, the RD of individual food items should probably be limited. Additionally, changes in ratios between food groups can be constrained. The restrictiveness of these constraints will inevitably limit the reductions in GHGE that can be achieved, and trade-offs may need to be made. The approach presented here could be applied not only for school settings but also for other sectors aiming to attain more sustainable procurement and planning of public meals.

Our findings show that it is possible to find nutritionally adequate solutions with only moderate (8–10%) average deviations from the observed food supply that comply with the 2030 Climate and Energy Framework of the European Commission and its goal of reducing the GHGE in the Region by 40% by 2030 [52]. A similar conclusion was reached by Milner et al. [53] who modelled a 40% decrease in GHGE of the average UK diet. Furthermore, in all solutions, the cost for the optimized food supply increased only moderately (by a maximum of 12% in Model 2 and a maximum of 2.6% in Model 3) or in many cases decreased, which is an important consideration for public meals where budgets are often tightly regulated.

Importantly, our study shows that considerable GHGE reductions can be achieved without omitting entire food categories. All optimized solutions were omnivorous; i.e., they included both plant and animal products such as eggs, milk or fish. This is an important aspect of cultural acceptability in Sweden, where the majority of the population consumes an omnivorous diet [54]. Other researchers aiming to align health and environmental priorities have recommended dietary approaches that exclude entire food categories, such as vegetarianism [55,56,57], based on the high contribution of livestock to the overall GHGE burden [58]. Although the overall mortality and incidence of non-communicable diseases decreases with an elevated intake of fruits and vegetables [59], vegetarian or vegan diets do not inevitably result in health improvement [60], and diets with appropriate shares of vegetables, fruits, pulses, meat and fish are also health-promoting [11,61]. Furthermore, the exclusion of an entire food category such as red meat could compromise nutritional status. Meat has a high bioavailability of iron and also enhances the absorption of iron from other foods [62]. Replacing meat and meat products with cereals, pulses, and tubers may negatively affect the iron status in vulnerable populations [62]. Current recommendations emphasize diversified diets with moderate shares of animal products as the most important strategy for achieving an adequate iron status [63].

In a recent review of studies assessing the sustainability of self-selected diets, several incompatibilities between health, affordability, and environmental dimensions were identified [13]. For example, in studies from France, diets with a higher nutritional quality were associated with higher GHGE [64,65] and higher cost [66]. Similarly, implementing food-based standards for English school meals aiming at improving nutritional quality was shown to result in increased GHGE [67]. Such findings suggest the need for a holistic approach where nutritional adequacy, affordability and acceptability are considered simultaneously [13].

As our aim was to develop such a holistic approach in which nutritional adequacy, affordability and acceptability are considered simultaneously [13], we adopted a comprehensive strategy where GHGE was first mathematically minimized while simultaneously integrating aspects of health, affordability and acceptability, in line with what others have done [23,24,25,68,69]. In addition, we showed that by also focusing on minimizing the deviation from the observed supply (as opposed to minimizing GHGE), and additionally constraining the relative deviation of foods to range between pre-determined limits, GHGE could still be reduced considerably but with less average deviation from the usual food supply and without the extreme deviations for individual food items that were the results of some of our initial models. Seemingly, with moderate GHGE reductions (up to 40%), a sufficiently large number of foods, as used by the school canteens over the school year, provides enough flexibility to Models 3 and 4 to keep the ARD values low. Similar to previous findings [23,26,27,49], the results from Models 3 and 4 suggest that this approach can achieve food supply patterns with low GHGE that are nutritionally adequate and that deviate only moderately from the current supply.

Constraining food-group ratios (as done in Model 4) has also previously been used to increase the acceptability of optimized diets [47,48,50]. In many cases, the selection of food-group ratios to control are made on an arbitrary basis [47,49]. In the current study, we applied an approach that included the ratios among all 15 food groups (105 in total), as even non-obvious relationships between food groups may affect the ability to plan the resulting school meals. However, preserving the food-group ratios in Model 4 resulted in a higher average relative deviation (ARD) of single foods from the observed compared to Model 3. Moreover, adding constraints on food-group ratios in Model 4 limited the ability to reduce GHGE compared to the other models, as seen in the case of School 3, where no solutions for a GHGE reduction below 10% were found, in which the RFGDs were limited to 20% or below.

Clearly, the more similar one requires the optimized diet to be to the baseline, the more one restricts the potential for reductions in GHGE. These are trade-offs that must be made by, or in conjunction with, the meal planner who also develops the final recipes. The goals of the optimization (i.e., whether health, environmental aspects, economic aspects, and/or similarity is prioritised), as well as the target population and the setting need to be taken into consideration when deciding on the parameters of the model. When optimizing the food supply, there is always an opportunity to assess the effects of the constraints applied to foods and/or food-group ratios and modify the applied constraints to tailor the model towards the priority of the user. For example, in this case, we allowed individual foods to deviate between −75%/+100% and −75%/+200%, but obviously these limits are flexible. Aiming for a 40% GHGE reduction using Model 3 resulted in increased shares of low climate impact food groups such as cereals, bread, pulses, and roots and tubers (Figure 2H), while the shares of higher climate impact groups such as dairy, poultry and red meat were reduced. These food group changes ranged between −50% and +60%, which consequently affected food-group ratios.

If, during the subsequent meal planning stage, changes in food-group ratios such as that of, for example, bread and solid fats are judged problematic for acceptability, applying constraints on the maximum allowed RFGD as in Model 4 is also possible [49,50]. Hence, to achieve the highest degree of acceptability, it may be necessary to involve meal planners and apply these models in an iterative process. As the individual observed food supply and preferences of the students or kitchen staff may vary across schools, no general rule on which specific ratios to apply can be recommended. The optimization may be re-run and adjusted (e.g., by changing the upper/lower limits for the deviation of individual foods as well as food-group ratios) according to the context-specific conditions [49].

### Limitations

The applied models did not take into account linkages between the production lines of foods; for example, the fact that beef and offal can be consumed as by-products of dairy farming. Offal and milk remained in the optimized supplies while meat was reduced. If these changes would occur at larger scale, the associated changes in consumer patterns may lead to a potential re-allocation of foods with high GHGE away from school canteens to other consumer groups. On a larger scale, particularly after taking market dynamics into consideration, this could lead to the inadequate usage of the entire animal and may therefore not result in the desired effect of a reduction in the environmental impact. Therefore, future studies should take the proportionalities among the parts of the slaughtered animals into consideration, along with the implementation of the share of beef that results from milk production, as done by Barré et al. [27].

Fish, often recommended as an environmentally friendly alternative to red meat [70], was one of the foods which increased considerably in the optimized food supply. Models 2 and 3 suggested increasing the supply of specific fish species (herring). It is also important here to consider external linkages, such as which other fish species are likely to be caught in the same net. Moreover, fish production from wild stocks cannot increase much, as 96% of the world’s fish stocks are already either moderately or fully exploited or over-fished [71]. Fish from even the lowest-impact aquaculture systems accounts for GHGE comparable to or even higher than that of poultry, pork and dairy and can be a source of eutrophication [72].

Other relevant aspects of food sustainability such as eco-toxicity, land use change, water use, eutrophication, acidification, animal welfare and biodiversity loss were not considered in the current study. However, data for these parameters are currently much more limited than those for GHGE, which can be used as a proxy for other environmental impact metrics [73].

The modelling of the optimized diets did not consider seasonality, although food purchases covered the whole school year. However, none of the foods reduced or increased after optimization was subjected to limited availability depending on the season (Figure S2). Moreover, buying locally produced foods according to season does not automatically imply lower GHGE, as these depend more on production systems (e.g., the types of inputs used and characteristics of production processes) rather than on country of origin [74]. The considerable variability in the environmental impacts of different production systems was not covered in the current study. The use of GHGE-data with improved accuracy for different ways of producing a food item would have given preference to the most climate-efficient production systems in the present models. However, that would have required more specific data from life-cycle analyses, which to date are not available for the Swedish context.

Although the number of schools was low, they came from different regions of Sweden (the east and south-west). The observed food supply of these schools was comparable to the nutritional quality of school meals today [75], and the solutions for each school were comparable. Our approaches did not include foods that were not already present in the buying lists, as they could potentially compromise acceptability (foods that pupils or school canteens are not familiar with). Future optimization studies might explore the inclusion of some of the many new foods emerging on the market with low GHGE in the model, such as oat- or algae-based products, or even include products fortified with important nutrients (i.e., those nutrients constraining the current solutions).

Finally, the energy needed (and thus GHGE) for the preparation of the optimized foods might have differed, but this was not possible to account for as further research is needed to provide such data.

## 5. Conclusions

A flexible strategy has been developed for optimizing food supplies for school canteens that can result in a supply that is considerably lower in GHGE, does not exclude any foods or food groups, and is nutritionally adequate and affordable by making only moderate changes to food amounts. Furthermore, if necessary, the strategy can preserve foods or ratios between food groups, which increases the likelihood of the result being acceptable. The priorities of the meal planning, whether aligning with health, environmental, economic, and/or cultural dimensions of diet sustainability, as well as the target population and setting need to be taken into consideration when deciding on the parameters of the model. Trade-offs between how much GHGE can be reduced and similarity may need to be made in the optimization of the school food supply. Additional minor adjustments may still be necessary at the meal planning stage, but this model could be an important tool for identifying how a food supply could be changed, and it should be of interest for meal planners and procurers. The application of the presented strategy is currently being evaluated in a real-world setting.

## Figures and Tables

**Table 1:** Characteristics of the four models applied to optimize the food supply. All models used the amounts of foods supplied as decision variables. All solutions provided by the models fulfilled the imposed set of nutritional constraints as provided in Supplemental Table S1.

Acronyms of Models	Objective Function (Minimum)	Climate Impact (CO_2_eq)	Affordability (Cost in SEK)	Constraints and Outputs	Constraints Applied to Achieve Higher Cultural Acceptability
Model 1: GHGE_min_^a^	GHGE ^b^	Minimized	Calculated	CO_2_eq minimized, RD constrained, ARD calculated	Individual food items’ RD progressively reduced, from 1000% until no feasible solution possible
Model 2: TRD_min_^c^	TRD	Progressively constrained by steps of 10% until no feasible solution possible	Calculated	TRD minimized, ARD and RFGC calculated	Individual food items’ RDs unconstrained (all food items could deviate unconditionally)
Model 3: CTRD_min_^d^	TRD	Progressively constrained by steps of 10% until no feasible solution possible	Calculated	TRD minimized, ARD, ARRD and RFGC calculated	Single food items’ RDs constrained to interval between an upper and a lower limit
Model 4: RTRD_min_^e^	TRD	Progressively constrained by steps of 10% until no feasible solution possible	Calculated	TRD minimized, ARD, ARRD and RFGC calculated	Single food items’ RDs and food-group ratios constrained to interval between an upper and lower limit

^a^ GHGE_min_, optimized for lowest achievable GHGE. ^b^ As total sum of CO_2_eq. ^c^ TRD_min_, optimized for minimum total relative deviation with unconstrained relative deviation for individual food items. ^d^ CTRD_min_, optimized for minimum total relative deviation with RD constrained for individual food items to range between an upper (positive) and a lower (negative) limit. ^e^ RTRD_min_, optimized for minimum total relative deviation with RD for individual food items and food-group ratios constrained to range between an upper (positive) and a lower (negative) limit. GHGE: greenhouse gas emissions; RD, relative deviation from observed food supply; SEK, Swedish krona, (1 SEK ≈ 0.104 United States dollar); TRD, total relative deviation; ARD, average relative deviation; ARRD, average relative ratio deviation (of food groups); RFGC, relative food group change.

**Table 2:** Effect of constraining GHGE, allowed relative deviations, and food-group ratio deviations (FGRD) on ARD and ARRD when minimizing TRD from the observed food supply in Models 2, 3, and 4.

					School 1		School 2		School 3	
Observed GHGE (CO_2_eq)					810 g		1022 g		967 g	
Model	CO_2_eq Reduction ^a^	Min RD	Max RD	Max FGRD	ARD	ARRD	ARD	ARRD	ARD	ARRD
	%	%	%	%	%	%	%	%	%	%
2 TRD_min_	na	na	na	na	1.5	39.7	2.5	32.2	2.9	42.3
	10	na	na	na	1.7	26.5	2.7	26.7	3.1	45.5
	20	na	na	na	2.0	17.7	3.0	28.8	3.2	46.8
	30	na	na	na	2.7	21.5	3.5	26.3	3.7	49.1
	40	na	na	na	2.7	21.5	4.2	41.1	4.6	62.8
	50	na	na	na	8.4	66.9	5.8	66.6	7.0	95.2
	60	na	na	na	15.4	144	8.9	114	11.4	165
	70	na	na	na	31.9	606	15.2	189	20.0	278
	80	na	na	na	78.1	950	24.5	410	34.5	278
	90	na	na	na	nfs	nfs	63.5	2360	70.3	1730
3 CTRD_min_	10	75	100	na	2.5	26.8	4.8	41.6	6.7	62.9
	10	75	200	na	2.0	24.3	3.7	46.6	3.8	66.2
	20	75	100	na	3.1	25.6	5.8	55.4	6.7	63.4
	20	75	200	na	2.4	21.5	4.2	47.6	4.0	61.9
	30	75	100	na	3.8	29.6	7.5	59.5	7.1	67.5
	30	75	200	na	4.8	41.7	4.9	57.9	4.7	68.7
	40	75	100	na	7.8	55.3	10.1	83.7	10.2	95.0
	40	75	200	na	8.7	63.6	6.8	83.0	7.8	63.8
	50	75	100	na	26.4	113	18.2	106	30.7	125
	50	75	200	na	19.8	112	11.1	101	18.8	94.0
	60	75	200	na	nfs	nfs	76.2	145	nfs	nfs
4 RTRD_min_	20	75	100	20	5.0	9.8	11.4	9.9	nfs	nfs
	20	75	100	10	6.4	5.0	14.2	4.8	nfs	nfs
	20	75	100	0	8.8	0.0	19.0	0.0	nfs	nfs
	20	75	200	20	3.5	8.1	6.8	8.5	nfs	nfs
	20	75	200	10	4.6	4.6	8.4	4.6	nfs	nfs
	20	75	200	0	6.3	0.0	10.5	0.0	nfs	nfs
	30	75	100	20	10.1	10.3	18.2	9.7	nfs	nfs
	30	75	100	10	14.4	4.7	24.1	5.0	nfs	nfs
	30	75	100	0	22.0	0.0	34.3	0.0	nfs	nfs
	30	75	200	20	7.5	10.3	9.8	9.4	nfs	nfs
	30	75	200	10	9.9	5.2	12.4	3.7	nfs	nfs
	30	75	200	0	13.4	0.0	15.7	0.0	nfs	nfs
	40	75	100	50	14.5	30.6	21.8	28.3	nfs	nfs
	40	75	100	40	20.1	24.1	28.0	21.7	nfs	nfs
	40	75	100	30	30.7	18.9	39.6	15.8	nfs	nfs
	40	75	100	20	nfs	nfs	nfs	nfs	nfs	nfs
	40	75	200	50	10.9	29.6	11.0	28.4	nfs	nfs
	40	75	200	40	13.2	23.3	12.9	21.7	nfs	nfs
	40	75	200	30	16.8	17.2	15.6	15.7	nfs	nfs
	40	75	200	20	22.8	11.8	20.0	10.0	nfs	nfs
	40	75	200	10	32.8	6.0	26.7	4.9	nfs	nfs
	40	75	200	0	75.4	0.0	38.2	0.0	nfs	nfs
	50	75	100	50	nfs	nfs	nfs	nfs	nfs	nfs
	50	75	200	50	60.6	35.8	32.9	33.5	nfs	nfs
	50	75	200	40	nfs	nfs	43.5	24.9	nfs	nfs
	50	75	200	30	nfs	nfs	74.0	18.0	nfs	nfs
	50	75	200	20	nfs	nfs	nfs	nfs	nfs	nfs

Relative reduction in carbon dioxide equivalents (CO_2_eq) per optimized food supply compared to baseline values. RD, relative deviation from observed food supply during the school year 2015/2016; ^a^ Relative reduction from observed CO_2_eq. ARD, average relative deviation from observed food supply during the school year 2015/2016 after optimization; ARRD, average relative ratio deviation (of food groups); GHGE, greenhouse gas emissions; TRD, total relative deviation. na, not applied; nfs, no feasible solution.

## Supplementary Materials

The following are available online at https://www.mdpi.com/1660-4601/16/17/3019/s1, Table S1: Nutrient constraints applied; Table S2: Amounts, cost, RDs, and GHGE of individual food items in the observed and optimized supply in Model 1 (GHGE_min_); Table S3: ARD, cost, and associated GHGE (CO_2_eq) when minimizing GHGE while applying constraints on the nutritional adequacy and maximum allowed RD from the observed food supply (Model 1, GHGE_min_); Table S4: Effect of constraining GHGE, allowed relative deviations, and food-group ratio deviations (FGRD) on the cost and number of foods removed, reduced and/or increased when minimizing TRD from the observed food supply in Models 2, 3, and 4; Figure S1: RD changes of individual food items when minimizing TRD (unconstrained) and constraining GHGE (Model 2); Figure S2: RD changes of individual food items when minimizing TRD (constrained to −75%/+200%), andconstraining GHGE (Model 3). A list of all foods used in the schools can be obtained from the corresponding author upon request. The CO_2_eq data can be obtained from RISE, Borås, Sweden (U.S.), upon individual agreement.
