# Supplementary material for: Optimizing School Food Supply: Integrating Environmental, Health, Economic, and Cultural Dimensions of Diet Sustainability with Linear Programming
Source: Int J Environ Res Public Health. 2019 Aug 21;16(17):3019. doi: 10.3390/ijerph16173019 (PMC6747157; doi:10.3390/ijerph16173019)
Supplement: Supplementary file 1 [file ijerph-16-03019-s001.pdf]

Table S1. Nutrient constraints applied during all linear optimization procedures.

Reference pupil – nutritional constraints for optimized food supply	Lower limit	Upper limit
**Energy (kcal)**[a]	604	604
**Carbohydrates (%E)**[b]	45	60
**Fat (%E)**[b]	22	40
**Protein (%E)**[b]	10	20
**Fiber (%E)**[c]	2	—
**Saturated fatty acids (%E)**[b]	—	10
**Mono unsaturated fatty acids (%E)**[b]	10	20
**Poly unsaturated fatty acids (%E)**[b]	5	10
**Vitamin A (µg)**[c]	168	—
**Vitamin D (µg)**[c]	3	30
**Vitamin E (mg)**[c]	2.2	90
**Thiamine (mg)**[c]	0.3	—
**Riboflavin (mg)**[c]	0.4	—
**Vitamin C (mg)**[c]	15.3	300
**Niacin (mg)**[c]	4.2	270
**Vitamin B6 (mg)**[c]	0.4	7.5
**Vitamin B12 (µg)**[c]	0.5	—
**Folate (µg)**[c]	57.6	300
**Phosphor (mg)**[c]	190.8	900
**Iodine (µg)**[c]	41.4	180
**Iron (mg)**[c]	3.3	7.5
**Calcium (mg)**[c]	246	750
**Potassium (mg)**[c]	810	—
**Magnesium (mg)**[c]	76.5	—
**Salt (g)**[d]	—	3.6
**Selenium (µg)**[c]	11.7	90
**Zink (mg)** [c]	2.7	7.5
**Omega-3 fatty acids (%E)**[c]	3.3	—

[a]Based on 30% of daily estimated energy requirement (EER), averaged values over 10 grades (preparatory class to ninth grade) and both sexes.
[b]Lower and upper limits based on 30% of daily recommended intake ranges, averaged values over 10 grades (preparatory class to ninth grade) and both sexes.
[c]Lower limit based on 30% of daily recommended intakes (RIs) and upper limit based on 30% of daily Upper intake levels (ULs), averaged values over 10 grades (preparatory class to ninth grade) and both sexes.
[d]Upper limit based on 30% of daily recommended intake (RIs).

Table S2. Amounts, cost, RDs, and associated GHGE (CO_2eq) of individual food items in observed and optimized supply, respectively, when minimizing GHGE and applying constraints on nutritional adequacy only (Model 1, $GHGE_{min}$).

	**School 1 (SEK 6.50[a])**				**School 2 (SEK 5.54[a])**				**School 3 (SEK 3.88[a])**			
**Food**	**Amount[b] (g)**		**Change[c] (RD, %)**	**CO_2eq[d] (g)**	**Amount[b] (g)**		**Change[c] (RD, %)**	**CO_2eq[d] (g)**	**Amount[b]**		**Change[c] (RD, %)**	**CO_2eq[d] (g)**
	Observed	Optimized			Observed	Optimized			Observed	Optimized		
Crisp bread	0.03	59.39	209156%	17.8								
Wheat bran									0.24	4.5	1777%	0.9
Semolina					0.86	55.24	6323%	24.3	0.55	17.95	3193%	7.9
Potato	22.90	133.43	483%	13.3	5.52	212.57	3748%	21.3				
Lentils dried									0.44	3.92	788%	1.3
Chickpeas dried	0.13	53.19	40338%	19.7	0.06	56.54	87254%	20.92				
Olive oil									0.15	3.61	2284%	7.4
Margarine 80%	0.53	2.35	341%	3.7	0.52	0.54	4%	0.85				
Parsley	0.05	42.27	91729%	14.8								
Carrots					1.29	11.89	818%	1.31	11.36	14.94	32%	1.6
Lingonberries									0.38	6.63	1652%	5.7
Anchovies									0.06	48.62	80149%	14.1
Pickled herring	0.063	20.65	32602%	12.8	0.08	5.2	6249%	3.2	0.04	0.94	49943%	11.7
Breaded herring	0.036	6.61	18511%	4.1								
Salt					0.52	0.06	-88%	0.02	0.04	0.22	480%	0.1
**Total**				**86 (10.6%)[e]**				**72 (7.0%)[e]**				**51 (5.3%)[e]**

[a]Cost of food supply per reference portion after optimization.
[b]Absolute raw amount of individual food items in observed and optimized supply, respectively.
[c]Relative change from observed food supply during the school year 2015/2016 after optimization.
[d]Amount of carbon dioxide equivalents (CO_2eq) of food supply after optimization.
[e]Percentage of observed GHGE
GHGE, Greenhouse gas emissions.
GHGE*min*, optimized for lowest achievable GHGE.

Table S3. ARD, cost, and associated GHGE (CO_2eq) when minimizing GHGE while applying constraints on nutritional adequacy and maximum allowed RD from observed food supply (Model 1, $GHGE_{min}$).

	ARD (%)			Cost[a] (SEK)			CO_2eq[b] (g)			# of food items Removed[c]			# of food items Reduced[c]			# of food items Increased[c]		
**School #**	**1**	**2**	**3**	**1**	**2**	**3**	**1**	**2**	**3**	**1**	**2**	**3**	**1**	**2**	**3**	**1**	**2**	**3**
**Observed value**				**9.07**	**10.63**	**11.22**	**810**	**1022**	**967**	**499**	**539**	**367**	**499**	**539**	**367**	**499**	**539**	**367**
**Max. RD per food (%)[d]**																		
Unconstrained[e]	887	293	480	6.50	5.54	3.88	86	72	51	492	532	358	0	1	0	7	6	9
1000	229	235	203	9.16	8.63	4.11	174	127	126	423	453	317	2	1	4	74	85	46
500	166	178	164	8.47	8.75	5.57	201	180	192	410	427	300	2	3	2	87	109	65
300	145	150	137	8.03	8.17	5.85	224	212	223	379	399	292	1	1	1	119	139	74
100	99.5	99.2	99.0	7.27	7.71	6.66	277	295	293	301	261	254	3	3	2	195	275	111
99	98.5	98.2	98.1	7.28	7.70	6.72	282	302	300	0	0	0	302	262	251	197	277	116
90	89.6	89.4	88.9	7.41	8.11	7.58	325	361	373	0	0	0	301	247	253	198	292	114
80	79.5	79.6	79.7	7.56	8.73	8.14	373	445	463	0	0	0	301	240	246	198	299	121
70	69.3	69.7	69.6	7.77	9.31	10.14	423	540	600	0	0	0	281	228	203	218	311	164
60	59.6	59.8	ns	7.97	9.73	ns	477	653	ns	0	0	ns	265	219	ns	234	320	ns
50	49.6	49.7	ns	8.28	10.44	ns	549	829	ns	0	0	ns	253	207	ns	246	332	ns
45	44.7	44.7	ns	8.73	10.92	ns	605	965	ns	0	0	ns	237	206	ns	262	333	ns
40	39.8	ns	ns	9.20	ns	ns	680	ns	ns	0	ns	ns	207	ns	ns	292	ns	ns

[a] Cost of food supply per reference portion after optimization

[b]Amount of carbon dioxide equivalents (CO_2eq) of food supply per reference portion after optimization.

[c]Number of foods removed, reduced or increased after optimization.

[d]Maximum allowed (negative or positive) relative deviation for individual food items from observed food supply during the school year 2015/2016.

[e]No constraint on maximum allowed (negative or positive) relative deviation individual food items from observed food supply during the school year 2015/2016.

ARD, Average relative deviation from observed food supply during the school year 2015/2016 after optimization.

GHGE, Greenhouse gas emissions.

GHGE*min*, optimized for lowest achievable GHGE.

RD, relative deviation from observed food supply during the school year 2015/2016.

SEK, Swedish Krona.

—, No achievable solution.

Table S4. Effect of constraining GHGE, allowed relative deviations, and food group ratio deviations (FGRD) on cost and number of foods removed, reduced and/or increased when minimizing TRD from observed food supply in Models 2, 3, and 4. na, not applied; ns, no solution; For other abbreviations, see legends and foot notes of Tables 1 and 2 and Tables S2 and S3.

					**School 1**				**School 2**				**School 3**			
**Observed cost**					**9.1 SEK**				**10.6 SEK**				**11.2 SEK**			
**Model**	**GHGE reduction**	**Min RD**	**Max RD**	**Max FGRD**	**Cost**	**Foods removed**	**Foods reduced**	**Foods increased**	**Cost**	**Foods removed**	**Foods reduced**	**Foods increased**	**Cost**	**Foods removed**	**Foods reduced**	**Foods increased**
	**%**	**%**	**%**	**%**	**SEK**				**SEK**				**SEK**			
2	na	na	na	na	9.3	4	2	3	10.7	4	1	4	11.7	2	7	2
	10	na	na	na	9.2	3	3	3	10.6	3	3	3	11.3	2	8	3
	20	na	na	na	8.9	5	3	3	10.5	4	3	3	11.0	2	8	3
	30	na	na	na	8.7	8	3	3	10.2	6	2	3	10.9	1	9	3
	40	na	na	na	8.7	8	3	3	9.7	8	2	3	10.4	1	13	4
	50	na	na	na	7.9	30	3	4	8.9	15	2	4	9.6	2	17	5
	60	na	na	na	7.7	58	3	4	8.4	28	3	3	8.8	3	24	5
	70	na	na	na	10.2	111	2	6	7.4	36	2	6	7.4	7	4	6
	80	na	na	na	10.1	176	1	8	6.6	74	3	6	6.2	10	63	4
	90	na	na	na	ns	ns	ns	ns	4.2	208	2	6	4.3	30	146	6
3	10	75	100	na	9.1	0	11	7	10.5	0	15	18	11.4	0	18	14
	10	75	200	na	9.1	0	10	3	10.6	0	13	7	11.4	0	19	14
	20	75	100	na	8.8	0	14	8	10.0	0	17	21	11.2	0	19	14
	20	75	200	na	8.9	0	13	3	10.4	0	12	9	10.3	0	34	14
	30	75	100	na	8.4	0	21	4	9.7	0	20	28	8.2	0	113	30
	30	75	200	na	8.5	0	22	10	9.7	0	15	11	11.5	0	12	4
	40	75	100	na	8.0	0	41	7	9.2	0	33	31	11.3	0	13	5
	40	75	200	na	8.0	0	43	14	9.3	0	25	11	10.9	0	15	5
	50	75	100	na	7.9	0	120	45	9.1	0	64	52	9.8	0	27	7
	50	75	200	na	7.8	0	98	16	8.6	0	46	16	8.3	0	73	9
	60	75	200	na	ns	ns	ns	ns	8.1	0	314	92	ns	ns	ns	ns
4	20	75	100	20	8.9	0	22	18	10.4	0	36	46	10.9	0	27	21
	20	75	100	10	8.8	0	29	22	10.2	0	45	53	ns	ns	ns	ns
	20	75	100	0	8.7	0	39	23	10.1	0	65	64	ns	ns	ns	ns
	20	75	200	20	8.7	0	20	8	10.3	0	26	16	ns	ns	ns	ns
	20	75	200	10	8.6	0	21	12	10.4	0	29	20	ns	ns	ns	ns
	20	75	200	0	8.6	0	26	13	10.2	0	37	21	ns	ns	ns	ns
	30	75	100	20	8.3	0	58	33	9.9	0	61	69	ns	ns	ns	ns
	30	75	100	10	8.4	0	85	50	9.9	0	85	79	ns	ns	ns	ns
	30	75	100	0	ns	ns	ns	ns	10.0	0	122	115	ns	ns	ns	ns
	30	75	200	20	8.2	0	40	17	10.0	0	32	25	ns	ns	ns	ns
	30	75	200	10	8.4	0	85	50	9.9	0	40	30	ns	ns	ns	ns
	30	75	200	0	8.5	0	72	29	9.9	0	40	30	ns	ns	ns	ns
	40	75	100	50	7.9	0	196	95	9.0	0	132	120	ns	ns	ns	ns
	40	75	100	40	ns	ns	ns	ns	ns	ns	ns	ns	ns	ns	ns	ns
	40	75	100	30	ns	ns	ns	ns	ns	ns	ns	ns	ns	ns	ns	ns
	40	75	100	20	ns	ns	ns	ns	ns	ns	ns	ns	ns	ns	ns	ns
	40	75	100	10	ns	ns	ns	ns	ns	ns	ns	ns	ns	ns	ns	ns
	40	75	100	0	ns	ns	ns	ns	ns	ns	ns	ns	ns	ns	ns	ns
	40	75	200	50	8.0	0	79	23	9.1	0	54	28	ns	ns	ns	ns
	40	75	200	40	7.9	0	104	30	9.1	0	63	32	ns	ns	ns	ns
	40	75	200	30	8.1	0	151	45	9.3	0	80	38	ns	ns	ns	ns
	40	75	200	20	ns	ns	ns	ns	9.3	0	112	46	ns	ns	ns	ns
	40	75	200	10	ns	ns	ns	ns	9.4	0	228	87	ns	ns	ns	ns
	40	75	200	0	ns	ns	ns	ns	ns	ns	ns	ns	ns	ns	ns	ns

		GHGE Unconstrained[1]			-10% GHGE			-20% GHGE			-30% GHGE			-40% GHGE			-50% GHGE		
		RD			RD			RD			RD			RD			RD		
		School 1	School 2	School 3	School 1	School 2	School 3	School 1	School 2	School 3	School 1	School 2	School 3	School 1	School 2	School 3	School 1	School 2	School 3
**Food group**	**Food subgroup**																		
Eggs	Eggs, raw/prepared		3 items, -100%	2 items, -100%		3 items, -100%	2 items, -100%		3 items, -100%	2 items, -100%		3 items, -100%	2 items, -100%		3 items, -100%	2 items, -100%		3 items, -100%	2 items, -100%
Other	Juice and nectar													-6%			-100%		
Solid fats and oils	Butter	-100%		1 item, -63%<br>1 item, -17%	-100%		-100%	-100%		-82%	-100%		-50%	-100%		-11%	-100%		1 item, -100%<br>1 item, +143%
	Margarine	-100%	-67%		-86%	-36%		-23%	-34%		+15%			+114%	+63%		+110%	+136%	
	Rapeseed oil		+33%	-100%			-100%			-100%			-100%			-98%			-100%
Fish	Salmon																		+128%
	White fish																+95%		-53%
Fruits and berries	Berries																		+171%
Vegetables<br>Roots and tubers<br>Pulses	Legumes			+321%			+380%			+429%			+475%			+496%			+468%
	Potatoes	+112%	+45%		+124%	+128%		+168%	+159%		+221%	+192%			+289%			+84%	
	Tomatoes, cucumbers, peppers																-100%		
Red meat<br>Poultry	Beef											1 item, -100%<br>1 item, -37%		-100%	2 items, -100%	-100%	-100%	2 items, -100%	2 items, -100%
	Meat balls/patties				-100%			2 items, -100%	-79%		4 items, -100%	-100%	-100%	4 items, -100%<br>1 item, -91%	-100%	2 items, -100%	8 items, -100%	2 items, -100%	2 items, -100%
	Meat dish																-100%		
	Minced meat					-60%	-52%		-100%	-94%		-100%	1 item, -100%<br>1 item, -71%		-100%	2 items, -100%		2 items, -100%	2 items, -100%
	Offal	+112%	+767%		+171%	+830%		+182%	+916%		+202%	+970%		+137%	+1021%		+89%	+867%	
	Pork																3 items, -100%	-100%	-100%
	Poultry													-11%	-44%		1 item, -100%<br>1 item, -1%	-100%	
	Sausages	-100%			-99%			-100%			2 items, -100%			2 items, -100%	-100%	-75%	3 items, -100%	1 item, -100%<br>1 item, -44%	2 items, -100%
	Vegetable dish with meat													-100%			2 items, -100%		
Other	Mayonnaise/dressing			-29%			-10%			-5%			-1%				-50%		-23%
	Salt	-29%		-20%	-29%		-17%	-27%		-16%	-18%		-13%			-17%			
Solid dairy<br>Other dairy	Cheese	-11%	2 items, -100%	-100%		2 items, -100%	-100%	-96%	2 items, -100%	-100%	-100%	2 items, -100%	-100%	1 item, -100%<br>1 item, -62%	2 items, -100%	-100%	5 items, -100%	3 items, -100%	-100%
	Cream	-100%	2 items, -100%	-100%	-100%	1 item, -100%<br>1 item, -94%	-100%	-100%	1 item, -100%<br>1 item, -31%	-100%	-84%	1 item, -100%<br>1 item, -75%	-100%	2 items, -100%	1 item, -100%<br>1 item, -95%	-100%	3 items, -100%	2 items, -100%	2 items, -100%
	Milk	+51%	+74%	+289%	+45%	+56%	+250%	-15%	+55%	+247%	-3%	+35%	+248%	-40%		+242%	-33%	1 item, -100%<br>1 item, -1%	+140%
Other	Nuts and seeds																		
Other	Sugar and sweets																		
Cereals<br>Bread	Crisp bread													+500%			+810%	+529%	
	Rice			-100%			-100%			-100%			-100%			-100%			-100%
	Flour or starch															-43%			

Figure S1. Summary of the RD for individual food items when minimizing TRD while applying constraints on nutritional adequacy and relative GHGE reductions (Model 2, TRD_{min}). [1]No constraint on maximum allowed amount of greenhouse gas emissions per meal; GHGE, Greenhouse gas emissions; RD, Relative deviation of individual food items from observed food supply after optimization, according to main food categories and food subcategories; TRD, Total relative deviation; TRD_{min}, optimized for minimum total relative deviation with unconstrained RD individual foods.

		GHGE Unconstrained[1]			-10% GHGE			-20% GHGE			-30% GHGE			-40% GHGE		
			RD			RD			RD			RD			RD	
		School 1	School 2	School 3	School 1	School 2	School 3	School 1	School 2	School 3	School 1	School 2	School 3	School 1	School 2	School 3
**Food group**	**Food subgroup**															
Eggs	Eggs, raw/prepared		3 items, +200%	2 items, +200%		3 items, +200%	2 items, +200%		3 items, +200%	2 items, +200%		3 items, +200%	2 items, +200%	+75%	3 items, +200%	2 items, +200%
Other	Juice and nectar													3 items, -75%		
Solid fats<br>Oils	Butter	-75%		-75%	-75%		1 item, -75%<br>1 item, -16%	-75%		1 item, -75%<br>1 item, +20%	-75%		1 item, -75%<br>1 item, +21%	-75%		1 item, -75%<br>1 item, +85%
	Margarine	-75%	-36%		-75%			-37%	+24%		+49%	+27%		+103%	+120%	
	Rapeseed oil			1 item, -75%<br>1 item, -51%		-48%	1 item, -75%<br>1 item, -56%		-75%	1 item, -75%<br>1 item, -51%		-75%	1 item, -75%<br>1 item, -52%	-8%	-75%	1 item, -75%<br>1 item, -60%
Fish	Salmon			+200%			+200%			+200%			+199%			+200%
	White fish													-17%		-75%
Fruits and berries	Berries												+64%			+174%
	Exotic fruits													-75%		
Vegetables<br>Roots and tubers<br>Pulses	Legumes		+200%	1 item, +200%<br>1 item, +168%		1 item, +200%<br>1 item, +85%	1 item, +200%<br>1 item, +123%		1 item, +200%<br>1 item, +114%	2 items, +200%		2 items, +200%<br>1 item, +190%	2 items, +200%		3 items, +200%<br>1 item, +190%	2 items, +200%
	Potatoes	+119%			+140%			+182%			+200%			+103%	+61%	
	Tomatoes, cucumbers, peppers													-75%		
	Tomatoes, processed															-75%
Red meat<br>Poultry	Beef											2 items, -75%	-75%	-75%	2 items, -75%	3 items, -75%
	Lamb															-75%
	Meat balls/patties				1 item, -75%<br>1 item, -34%	-62%		3 item, -75%<br>1 item, -12%	-75%	-50%	5 item, -75%<br>1 item, -40%	-75%	2 items, -75%	8 items, -75%	3 items, -75%	3 items, -75%
	Meat dish													-75%		
	Minced meat		+79%	-46%					-60%	-75%		-75%	2 items, -75%		2 items, -75%	2 items, -75%
	Offal	+159%	+200%		+170%	+200%		+187%	+200%		+192%	+200%		+111%	+200%	
	Pork													2 items, -75%	1 items, -75%<br>1 item, -25%	-75%
	Chicken		-2%						-47%		-75%	-75%		2 items, -75%	-75%	
	Vegetable dish with meat	-75%	1 item, -75%<br>1 item, +87%	-75%	-75%	-75%	-75%	2 items, -75%	-75%	-75%	2 items, -75%	-75%	-75%	3 items, -75%	2 items, -75%<br>1 item, -73%	3 items, -75%<br>1 item, -71%
											-75%			2 items, -75%		
Other	Mayonnaise/dressing			-75%			-75%			-75%			-75%	-75%		-75%
	Salt	-33%		-16%	-28%		-18%	-23%		-15%	-20%		-9%			
	Ketuchup, mustard		+200%			+200%			+200%			+200%		-75%	+200%	
Solid dairy<br>Other dairy	Cheese	2 items, -75%	4 items, -75%	-75%	1 item, -75%<br>1 item, -72%	3 items, -75%<br>1 item, -55%	-75%	2 items, -75%	3 items, -75%	-75%	4 items, -75%	3 items, -75%	-75%	6 items, -75%	4 items, -75%	2 items, -75%
	Cream	3 items, -75%	3 items, -75%	1 item, -75%<br>1 item, -70%	2 items, -75%	3 items, -75%	2 items, -75%	2 items, -75%	2 items, -75%<br>1 item, -57%	2 items, -75%	3 items, -75%	2 items, -75%	-75%	3 items, -75%<br>1 item, -57%	3 items, -75%	2 items, -75%
	Milk	+39%	1 item, -75%<br>1 item, +46%	1 item, +200%<br>1 item, +68%	+39%	+37%	1 item, +200%<br>1 item, +119%	+21%	+25%	+3%	1 item, -56%<br>1 item, -3%	+25%	+200%	-35%		+168%
	Yoghurt and sour milk		-75%			-75%			-75%			-61%			-75%	-75%
Other	Nut or seed product	-34%														
Cereals<br>Bread	Crisp bread					+200%			+200%		+68%	+200%		2 items, +200%	1 item, +200% 1 item, +127%	
	Breakfast cereals		+200%			+200%			+200%	+8%		+200%	+17%		+200%	
	Flour or starch					-22%							-16%			-22%
	Grains and bran					+200%			+200%			+200%			+200%	
	Pasta, bulgur, couscous etc.								+200%			+200%		+200%	+200%	
	Rice		-75%	-75%		-75%	-75%		-75%	-75%		-67%	-75%	-75%	-75%	-75%

Figure S2. Summary of the RD for individual food items when minimizing TRD while applying constraints on nutritional adequacy, relative GHGE reductions, and additional constraints on the RD of individual food items (Model 3, CRD_{min}).[1]No constraint on maximum allowed amount of carbon dioxide equivalents (CO_2eq) per optimized food supply; GHGE, Greenhouse gas emissions; TRD, Total relative deviation; RD, Relative deviation of individual food items from baseline food supply after optimization, according to main food categories and food subcategories; CRD_{min}, optimized for minimum total relative deviation while constraining the relative deviation of individual food items to a range between -75% and +200%.
